# Isolated growth hormone deficiency in children with vertically transmitted short stature: What do the genes tell us?

**DOI:** 10.3389/fendo.2022.1102968

**Published:** 2023-01-13

**Authors:** Lukas Plachy, Shenali Anne Amaratunga, Petra Dusatkova, Klara Maratova, Vit Neuman, Lenka Petruzelkova, Dana Zemkova, Barbora Obermannova, Marta Snajderova, Stanislava Kolouskova, Zdenek Sumnik, Jan Lebl, Stepanka Pruhova

**Affiliations:** Department of Pediatrics of Second Faculty of Medicine Charles University in Prague and Motol University Hospital, Prague, Czechia

**Keywords:** short stature, growth hormone, growth hormone deficiency, genetics, next-generation sequencing

## Abstract

**Introduction:**

The growth hormone deficiency (GHD) diagnosis is controversial especially due to low specificity of growth hormone (GH) stimulation tests. It is therefore believed that children diagnosed with GHD form a heterogeneous group with growth disorder frequently independent on GH function. No study evaluating the complex etiology of growth failure in children with diagnosed GHD has been performed thus far.

**Aims:**

To discover genetic etiology of short stature in children with diagnosed GHD from families with short stature.

**Methods:**

Fifty-two children diagnosed with primary GHD and vertically transmitted short stature (height SDS in the child and his/her shorter parent <-2 SD) were included to our study. The GHD diagnosis was based on growth data suggestive of GHD, absence of substantial disproportionality (sitting height to total height ratio <-2 SD or >+2 SD), IGF-1 levels <0 for age and sex specific SD and peak GH concentration <10 ug/L in two stimulation tests. All children were examined using next-generation sequencing methods, and the genetic variants were subsequently evaluated by American College of Medical Genetics standards and guidelines.

**Results:**

The age of children at enrollment into the study was 11 years (median, IQR 9-14 years), their height prior to GH treatment was -3.0 SD (-3.6 to -2.8 SD), IGF-1 concentration -1.4 SD (-2.0 to -1.1 SD), and maximal stimulated GH 6.3 ug/L (4.8-7.6 ug/L). No child had multiple pituitary hormone deficiency or a midbrain region pathology. Causative variant in a gene that affects growth was discovered in 15/52 (29%) children. Of them, only 2 (13%) had a genetic variant affecting GH secretion or function (*GHSR* and *OTX2*). Interestingly, in 10 (67%) children we discovered a primary growth plate disorder (*ACAN*, *COL1A2*, *COL11A1*, *COL2A1*, *EXT2*, *FGFR3*, *NF1*, *NPR2*, *PTPN11* [2x]), in one (7%) a genetic variant impairing IGF-1 action (*IGFALS*) and in two (12%) a variant in miscellaneous genes (*SALL4*, *MBTPS2*).

**Conclusions:**

In children with vertically transmitted short stature, genetic results frequently did not correspond with the clinical diagnosis of GH deficiency. These results underline the doubtful reliability of methods standardly used to diagnose GH deficiency.

## Introduction

The correct production, secretion, and function of growth hormone (GH) is important for the physiological growth and optimal functioning of the human organism (1, 2). For people with growth hormone deficiency (GHD), treatment with recombinant GH is essential to achieve normal adult height and, in cases of severe GHD, prevent repeated episodes of hypoglycaemia or other possible consequences of impaired metabolic GH function (1, 3). Precise diagnosis of individuals with GHD allowing early GH treatment is therefore crucial (2).

The diagnosis of GHD is complex combining auxological, laboratory and radiological examination. Growth hormone stimulation tests are performed for the confirmation of the diagnosis (3, 4). However, these tests are known to have low specificity, potentially causing false positive results (5–8). Consequently, children diagnosed with GHD likely form a rather heterogeneous group with different etiology of growth disorder frequently independent of GH production or function (9). However, no studies evaluating the complex genetic etiology of growth failure in children that have been clinically classified as having GHD have been performed so far.

Importantly, modern genetic methods including next-generation sequencing (NGS) have shown their potential to discover the causes of growth disorders on a molecular basis (9, 10). The boom in genetic diagnostics in the last two decades has led to a substantial progress in understanding the etiology of short stature (9, 11). In our study, we aimed to search for genetic background of short stature in children diagnosed as GHD from families with short stature.

## Materials and methods

### Patients

#### Inclusion criteria

According to the records database, 747 children are currently treated with GH in our center. After excluding children with Prader-Willi syndrome, Turner syndrome, and those with known secondary cause of their growth disorder (e.g., chronic kidney disease, secondary GHD caused by intracranial tumor, surgery and/or irradiation), 528 patients remained for further evaluation. Within this group, 419 individuals were diagnosed with primary GHD. Out of these, 70 had vertically transmitted short stature defined as a height SDS ≤-2 SD in both the child and his/her shorter parent and therefore, were chosen for the study. A total of 52 study participants/their legal guardians signed written informed consent before genetic examination and were included in the study. The study was approved by the institutional Ethics Committees of the 2^nd^ Faculty of Medicine (approval number EK-753.3.5/21) of Charles University in Prague, Czech Republic.

#### Clinical evaluation

The heights and body proportionality (sitting height to total height [SHH] ratio) of all participants were obtained during anthropometric measurements. Data regarding birth parameters were obtained from the medical records. The parents’ heights were measured to the nearest 1 mm and the heights of other relatives were obtained from the parents. All the data was standardized according to recent normative values (12–14). Bone age was evaluated using the Tanner-Whitehouse method (15).

#### Diagnostics of growth hormone deficiency

Growth hormone deficiency was diagnosed using current guidelines (3, 4). In all children with auxological data suggestive of GHD (i.e., current height <-3 SD below the mean, current height <-1.5 SD below the midparental height and/or current height <-2 SD below the mean combined with a decrease in height >0.5 SD over one year in a child older than 2 years), IGF-1 levels <0 SD (reference ranges standardized for sex and age) and no substantial disproportionality (SHH ratio <-2 SD or >+2 SD) GH stimulation tests were performed. Children with a maximum GH concentration <10 ug/L in both the clonidine and insulin hypoglycemia tests were classified as having GHD. Sex-steroid priming was performed in children aged 9 years and older.

### Genetic testing

Genomic DNA was extracted from peripheral blood (QIAamp Blood Mini Kit, Quiagen, Hilden, Germany) in all children included in the study. Firstly, some children underwent basic genetic testing. Turner syndrome and SHOX haploinsufficiency were examined in all girls by fluorescence *in situ* hybridization (FISH). In children with a clinical suspicion of a specific genetic disorder, targeted genetic testing was performed. Children with no genetic cause of short stature elucidated at this point were subsequently examined using the NGS methods: whole-exome sequencing (WES) or custom-targeted NGS panel containing 398 genes associated with growth (**Supplementary Table 1**). All variants from the NGS were confirmed by Sanger sequencing as we described previously (16). The method of genetic examination we described in detail in previous studies (11, 17, 18).

All variants with potential clinical importance were evaluated by American College of Medical Genetics and Genomics (ACMG) standards and guidelines (19). For variant evaluation, we also used the ACMG criteria implemented into the VarSome software (20) and Franklin software (https://franklin.genoox.com version date 2^nd^ November 2022) that score each ACMG rule as very strong, strong, moderate, or supporting based on ACMG recommendations and its more up-to-date modifications. In some cases, the strength of the rules was modified according to extended investigation of various databases and clinical evaluation of the patient. To evaluate the segregation of genetic variants with short stature in the families, DNA and height data of other relatives was obtained. The guidelines formulated by Jarvik et al. were followed (21) and applied to co-segregation in the pathogenicity classification. At the end, all genetic variants were classified as pathogenic (P), likely pathogenic (LP), benign (B), likely benign (LB) or as variants of uncertain significance (VUS).

## Results

In total, 52 children with a primary GHD diagnosis and vertically transmitted short stature were enrolled to the study. Their age at study enrolment was 11 years (median; IQR 9-14 years), their pretreatment height was -3.0 SD (-3.6 to -2.8 SD), their shorter parent’s height was -2.6 SD (-2.9 to -2.2 SD), their IGF-1 concentration prior to the GH treatment was -1.4 SD (-2.0 to -1.1 SD),their stimulated GH concentration maximum was 6.3 ug/L (4.8-7.6 µg/L), and their bone age was delayed by 1.1 years (0.3-1.7 years). Seventeen children had mild GHD with stimulated GH concentrations of 7.0-9.9 µg/L, 34 children had stimulated GH concentrations 3.0-6.9 µg/L and only one child had severe GHD with stimulated GH concentration <3.0 µg/L. No child had multiple pituitary hormone deficiency or a midbrain region pathology on magnetic resonance imaging. The birth length and birth weight of the children in the study cohort was -1.8 SD (median; IQR -2.4 to -1.2 SD) and -1.3 SD (-2.0 to -0.7 SD), respectively. Twenty-two children were born small for gestational age (SGA) (9 for both birth length and weight, 11 only for birth length, and 2 only for birth weight). The children have been treated with GH for a median 5.0 years (3.5-6.0 years), with a median dose of 32 µg/kg/day (30-34 µg/kg/day) during the first year of treatment.

A monogenic cause of growth failure was elucidated in 15 (29%) of 52 children with diagnosed primary GHD and vertically transmitted short stature who were enrolled to the study. Of them, only 2 (13%) had a genetic variant affecting GH secretion or function (*GHSR* and *OTX2*). Interestingly, 10/15 (67%) children had a genetically proven primary growth plate disorder (4 had extracellular matrix protein defect [genes *ACAN*, *COL1A2*, *COL11A2*, and *COL2A1*], 2 had impaired paracrine regulation of the growth plate [genes *NPR2*, and *FGFR3*], and 4 had a disorder affecting fundamental intracellular processes of the growth plate [*PTPN11* gene in 2 patients, and *EXT2* and *NF1* genes each in a single patient]. Among the remaining children, 1/15 (7%) had altered IGF function (gene *IGFALS*), and 2/15 (13%) had mutations in miscellaneous genes (*MBTPS2* and *SALL4*). Specific genetic variants and phenotypes of the children are summarized in **Table 1**, data evaluating children with genetically confirmed GHD and those with genetically diagnosed primary growth plate disorder are summarized in **Table 2**.

**Table 1:** A table evaluating clinical characteristics and genetic examination results in children with proven monogenic aetiology of their short stature.

Patient	Sex	Age at last follow-up (years)	GH treatment initiation age (years)	GH dose in the first year of GH treatment (ug/kg/day)	Shorter parent's height (SD)	Birth weight (SD)	Birth length (SD)	Height SDS prior to GH treatment	Height SDS after 1 year of GH treatment	Height SDS after 3 years of GH treatment	Growth velocity prior to GH treatment (cm/year)	Growth velocity in the first year of GH treatment (cm/year)	BA prior to GH treatment (difference to CA. years)	IGF1 prior to GH treatment (SD)	Stimulated GH maximum (ug/l)	SHH ratio (SD)	Additional phenotypic features	Genetic examination method	Gene	Variant type	Transcript variant	Protein variant	Classification	ACMG criteria
Impaired growth hormone production																								
1	F	10	7	31	-2.8	-2.6	-3.1	-3.2	-2.1	-1.4	4.2	10.6	-0.6	-2.1	7.2	0.1	Mother HELLP syndrome in pregnancy	WES	GHSR	M/n	c.526G>A	p.Gly176Arg	LP	PM2(m), PP1(m), PP3(m)
2	M	10	6	28	-2.8	-1.2	-1.1	-6.3	-5.0	-3.4	4.5	12.1	0.0	<-3	1.7	0.4	Psychomotor retardation	NGS panel	OTX2	M/n	c.106delC	p.Arg36fs	P	PVS1(vs), PM2(m), PP5(sp)
Alteration of IGF function																								
3	M	11	7	34	-2.8	-1.6	-1.7	-3.5	-2.6	-1.6	5.3	9.8	-0.2	-1.8	6.1	0.6	–	WES	IGFALS	M/n	c.589C>T	p.Arg197Cys	LP	PP1(st), PM2(m)
Primary growth plate disorder																								
Extracellular matrix																								
4	M	14	7	30	-3.6	-1.6	-3.3	-3.7	-2.9	-2.2	3.7	8.6	1.7	-1.6	3.2	0.9	–	Sanger	ACAN	M/n	c.1425del	p.Val478fs*14	P	PVS1(vs), PM2(m), PP1(st)
5	M	12	5	35	-2.0	-2.5	-1.5	-3.2	-2.3	-1.9	3.8	9.9	-2.0	-0.9	6.4	-0.2	Frequent long bone fractures, vertebrae compressive fractures	NGS panel	COL1A2	M/n	c.577G>A	p.Gly193Ser	P	PM2(m), PM6(m), PP2(sp), PP3(st), PP4(sp), PP5(s)
6	M	16	10	35	-2.0	-1.0	-0.7	-3.4	-2.7	-2.1	3.8	7.8	-1.3	-1.2	8.0	0.7	–	NGS panel	COL11A2	M/n	c.3706C>T	p.Arg1236Cys	LP	PM2(m), PP1(st), PP3(sp)
7	M	16	12	33	-2.9	-2.4	-3.0	-3.4	-2.9	-2.2	4.1	8.7	-1.5	-1.6	8.4	1.2	–	WES	COL2A1	M/n	c.3016C>G	p.Arg1036Gly	LP	PM1(sp), PP1(m), PP2(sp), PP3(sp)
Paracrine growth plate regulation																								
8	F	5	4	35	-2.4	-1.6	-2.4	-3.0	-2.9	NA	3.7	6.7	NA	-2.1	4.0	0.3	–	NGS panel	FGFR3	M/n	c.251C>T	p.Ser84Leu	LP	PM2(m), PP1(sp), PP2(sp), PP5(st)
9	M	9	7	32	-2.4	0.9	-1.7	-2.9	-2.2	NA	5.6	10.3	-1.7	-1.4	4.7	0.3	–	NGS panel	NPR2	M/n	c.613C>T	p.Arg205*	P	PVS1(vs), PM2(m), PP3(sp)
Fundamental Intracellular processes of the growth plate																								
10	M	12	8	33	-2.9	-2.4	-3.4	-2.7	-2.0	-1.3	4.6	9.0	0.5	-1.0	5.9	0.5	–	WES	EXT2	M/n	c.2034C>G	p.Asn678Lys	LP	PM2(m), PP1(st), PP3(sp)
11	M	16	11	33	-2.9	1.0	-1.2	-3.9	-2.9	-2.6	2.5	9.2	0.5	-4.3	6.2	0.7	Vitiligo	Sanger	PTPN11	M/n	c.211T>A	p.Phe71Ile	LP	PM1(m), PM2(m), PM5(m), PP2(sp), PP3(sp)
12	M	12	7	33	-2.6	NA	NA	-2.7	-1.9	-1.2	4.0	9.3	-1.6	-1.4	4.8	1.5	–	Sanger	PTPN11	M/n	c.1403C>T	p.Thr468Met	P	PS3(st), PM1(sp), PM2(m), PM5(m), PP2(sp), PP3(sp), PP5(m)
13	F	12	6	33	-3.9	1.0	0.1	-2.5	-1.7	-1.0	4.8	8.7	-0.7	-1.1	1.2	NA	Café-au-lait spots, hamartomas in the brain	Sanger	NF1	M/n	c.4267A>G	p.Lys1423Glu	P	PS3(st), PM1(m), PM2(m), PM5(m), PP2(sp), PP3(sp), PP5(vs)
Genes with miscellaneous function																								
14	M	10	5	34	-2.7	-1.1	-1.8	-4.4	-4.0	-3.6	5.9	7.2	NA	-1.1	6.4	0.9	Immunodeficiency, Hyperkeratosis, cornea scarring	WES	MBTPS2	M/-	c.1538T>C	p.Leu513Pro	LP	PM1(sp), PM2(m), PP3(m), PP5(sp)
15	M	12	7	34	-2.4	-1.8	-1.4	-2.8	-2.1	-1.0	6.3	8.7	NA	-1.0	4.6	-0.3	Radial ray defect. right kidney dystopia	WES	SALL4	M/n	c.1717C>T	p.Arg573*	P	PVS1(vs), PM2(m), PP1(sp), PP4(sp), PP5(sp)

ACMG, American College of Medical Genetics guidelines; BA, bone age; CA, calendar age; F, female; GH, growth hormone; LP, likely pathogenic; M, male; (m), moderate strength of the criterion used; M/- - hemizygote, M/M, homozygote, M/n, heterozygote, NA, not available; NGS, next-generation sequencing; P, pathogenic; SD, standard deviation; (sp), supporting strength of the criterion used; (st), strong strength of the criterion used; SHH, sitting height to height; (vs), very strong strength of the criterion used; WES, whole exome sequencing.

**Table 2:** Table evaluating clinical data in children with genetically confirmed GH deficiency and those with genetically diagnosed primary growth plate disorder.

	Patient 1 with genetically confirmed GHD	Patient 2 with genetically confirmed GHD	Patients with genetically diagnosed primary growth plate disorder
**Gene found**	*GHSR*	*OTX2*	NA
**Shorter parent’s height (SD)**	-2.8	-2.8	-2.8 (-2.9 to -2.4)
**IGF-1 prior to GH treatment (SD)**	-2.1	<-3.0	-1.4 (-1.6 to -1.1)
**Maximal stimulated GH concentration (ug/L)**	7.2	1.7	5.4 (4.0 to 6.4)
**Height prior GH treatment (SD)**	-3.2	-6.3	-3.1 (-3.4 to -2.7)
**Height after one year of GH treatment (SD)**	-2.1	-5.0	-2.5 (-2.9 to -2.0)
**Height after 3 years of GH treatment (SD)**	-1.4	-3.4	-2.0 (-2.2 to -1.3)
**Growth velocity prior to GH treatment (cm/year)**	4,2	4,5	3.9 (3.7 to 4.6)
**Growth velocity in the first year of GH treatment (cm/year)**	10,6	12,1	8.9 (8.6 to 9.3)

The values in children with genetically diagnosed primary growth plate disorder are expressed as medians and interquartile ranges. GH, growth hormone; NA, not applicable; SD, standard deviation.

## Discussion

In our study, we evaluated the complex genetic etiology of growth disorders in children with diagnosed primary GH deficiency from families with short stature. Interestingly, GHD was genetically confirmed as a cause of growth failure only in a minority of children (13% of children with genetic etiology discovered, 4% of the whole study cohort). On the other hand, modern genetic examination methods frequently discovered other mechanisms causing growth disorders independent of GH production, secretion or function, further broadening the doubts about the GHD diagnostics.

Studies evaluating the genetic etiology in children classified as GHD are scarce. Depending on the cohort, causative genetic variants were found in approximately 11% of children with primary isolated GHD (22). In the remaining majority of children with diagnosed IGHD, specific causes of their short stature are unknown. These children are traditionally labelled with a descriptive diagnosis of an “idiopathic growth hormone deficiency” (9) and the etiology of their growth disorder remains to be elucidated. In our study, we found genetic causes of GHD in even smaller proportion of children (2/52; 4%). However, unlike in the previous studies, we focused not only on genes causing GHD, but on other possible genetic causes of short stature as well. Using this strategy, we discovered the genetic etiology of growth failure in an additional 25% (13/52) of children. Surprisingly, 67% (10/15) of them had a primary growth plate disorder.

The discrepancy between the original clinical diagnosis of GHD and the genetic finding in most cases raises a question of which method is more accurate. Importantly, current diagnostics of GHD faces many difficulties and is considered as one of the most controversial issues in pediatric endocrinology (5). Growth hormone stimulation tests currently used as golden standard of the GHD diagnosis (4) are not physiological and have a very low specificity and reproducibility. Furthermore, they are affected by pubertal development, obesity, or other characteristics of the examined individual (3, 5). Unfortunately, there is no method reliably proving GHD to validate GH stimulation tests (5). A recent study by Bright et al. calculated an extremely low probability (2.8%) of a true-positive GH stimulation test in a child with short stature (23). Due to all these reasons, it is believed that most children with diagnosed GHD have a non-pituitary etiology of their short stature and are erroneously labelled as GHD (9). The results of our study support this presumption and offers the first insight into the possible non-pituitary causes of growth disorders in such children.

On the other hand, in the absence of a validation method for GHD diagnostics (5), there is no way to prove the diagnosis of GHD is incorrect. We must therefore admit the possibility that GHD might contribute to the patients’ short stature in additional to the cause discovered by genetic testing. To correctly interpret the results of our study, we need to consider the possibility of false positive genetic results especially if the likely pathogenic variants are considered causative. However, the probability of a likely pathogenic variant to be truly pathogenic is >90% (19) and are therefore more trustworthy than the methods traditionally used to diagnose GHD.

Our study had several strengths. Firstly, we examined a homogenous population of patients from a single center of an economically stable country with high quality health care, low consanguinity rate, and negligible social causes of growth failure such as malnutrition. Secondly, all GH stimulation tests were performed by experienced investigators using a defined protocol and the results were analyzed in a single laboratory using the same methodology. However, our study had several limitations as well. Firstly, functional studies were not performed in our study. However, according to ACMG standards and guidelines, the causality of genetic variants can be proven also by other methods (19). In our study evaluating children with vertically transmitted short stature, the segregation of genetic variants in short people within the families was an important factor. Secondly, protein non-coding variants (in the exception of disruption in the exon-intron boundaries) were not captured by the NGS. Thirdly, copy number variants were not evaluated in the current study. Fourthly, although our NGS panel included a large number of genes associated to growth disorders (398), causative variants in the genes not present in the panel could have been missed. Moreover, children with known genetic cause of their short stature prior to the study were not examined using NGS. Finally, only children with vertically transmitted short stature were included in our study. To generalize these results to all children with diagnosed GHD, further studies are warranted.

## Conclusion

In children with vertically transmitted short stature, genetic results frequently did not correspond with the clinical diagnosis of GH deficiency. These results underline the doubtful reliability of methods standardly used to diagnose GH deficiency.

## Data availability statement

The datasets presented in this article are not readily available for ethical and legal reasons relating to the participants’ privacy rights. The raw sequencing data are available upon reasonable request to the corresponding author (lukas.plachy@fnmotol.cz).

## Ethics statement

The studies involving human participants were reviewed and approved by Ethics Committees of the 2nd Faculty of Medicine (approval number EK-753.3.5/21) of Charles University in Prague, Czech Republic. Written informed consent to participate in this study was provided by the participants’ legal guardian/next of kin.

## Author contributions

LuP organized the study, contributed to the study design of the study, helped to obtain clinical data, contributed to the interpretation of the genetic results, contributed to the results interpretation, wrote the manuscript. SA contributed to the study design of the study, contributed to the results interpretation, contributed to the interpretation of the genetic results, contributed to the final version of the manuscript. PD contributed to the study design of the study, performed the genetic examination, supervised the interpretation of genetic results, contributed to the results interpretation, contributed to the final version of the manuscript. KM, DZ contributed to the study design of the study, performed antropometric measurements, contributed to the results interpretation, contributed to the final version of the manuscript. VN, LeP, BO, MS, SK, ZS, JL contributed to the study design of the study, helped to obtain clinical data, contributed to the results interpretation, contributed to the final version of the manuscript SP. Supervised the whole study, contributed to the study design of the study, helped to obtain clinical data, contributed to the genetic results interpretation, contributed to the results interpretation, contributed to the final version of the manuscript. All authors contributed to the article and approved the submitted version.
