# Supplementary material for: Isolated growth hormone deficiency in children with vertically transmitted short stature: What do the genes tell us?
Source: Front Endocrinol (Lausanne). 2023 Jan 13;13:1102968. doi: 10.3389/fendo.2022.1102968 (PMC9880029; doi:10.3389/fendo.2022.1102968)
Supplement: Supplementary file 1 [file Presentation_1.pptx]

## Slide 1
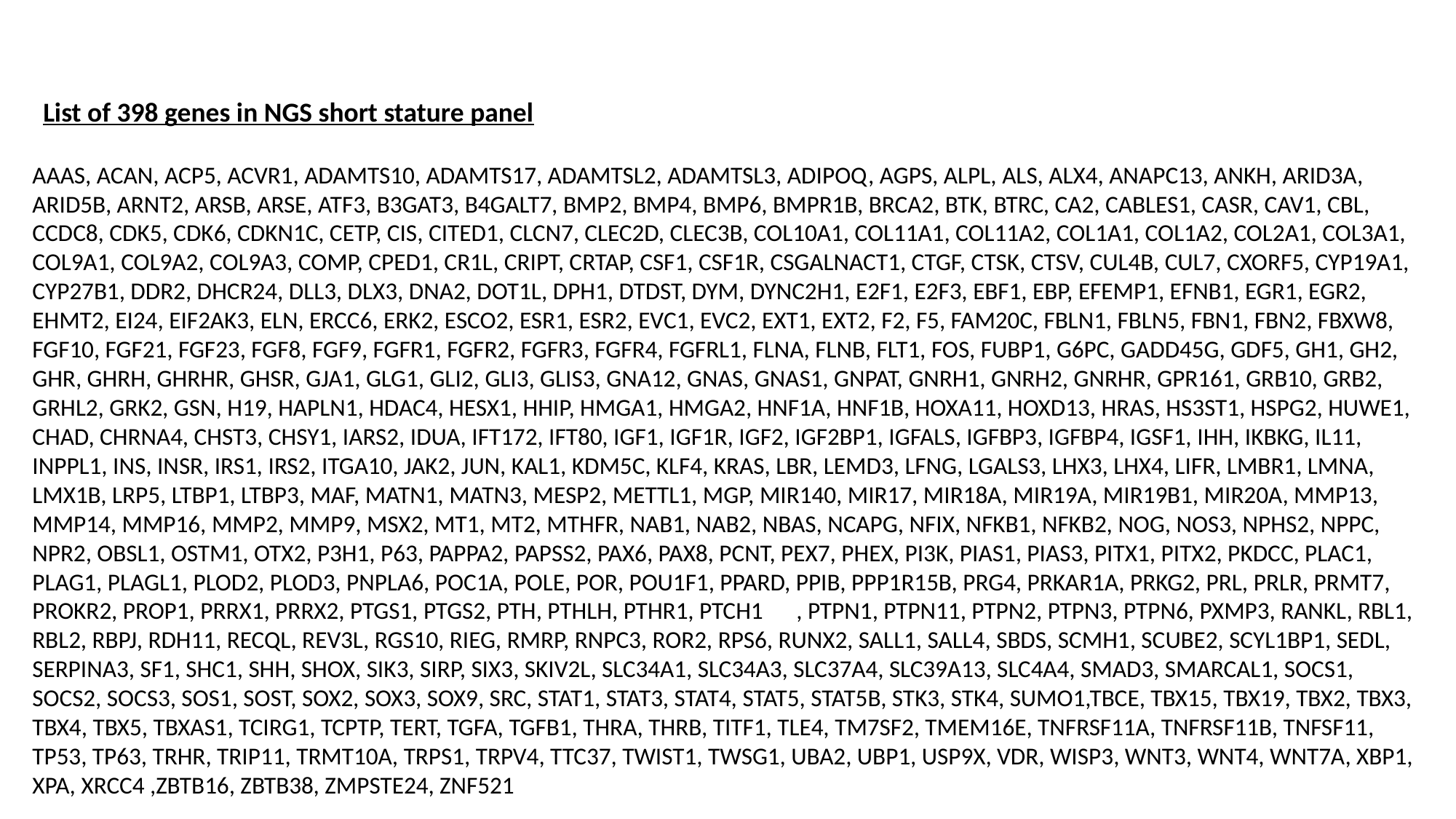

List of 398 genes in NGS short stature panel
AAAS, ACAN, ACP5, ACVR1, ADAMTS10, ADAMTS17, ADAMTSL2, ADAMTSL3, ADIPOQ, AGPS, ALPL, ALS, ALX4, ANAPC13, ANKH, ARID3A, ARID5B, ARNT2, ARSB, ARSE, ATF3, B3GAT3, B4GALT7, BMP2, BMP4, BMP6, BMPR1B, BRCA2, BTK, BTRC, CA2, CABLES1, CASR, CAV1, CBL, CCDC8, CDK5, CDK6, CDKN1C, CETP, CIS, CITED1, CLCN7, CLEC2D, CLEC3B, COL10A1, COL11A1, COL11A2, COL1A1, COL1A2, COL2A1, COL3A1, COL9A1, COL9A2, COL9A3, COMP, CPED1, CR1L, CRIPT, CRTAP, CSF1, CSF1R, CSGALNACT1, CTGF, CTSK, CTSV, CUL4B, CUL7, CXORF5, CYP19A1, CYP27B1, DDR2, DHCR24, DLL3, DLX3, DNA2, DOT1L, DPH1, DTDST, DYM, DYNC2H1, E2F1, E2F3, EBF1, EBP, EFEMP1, EFNB1, EGR1, EGR2, EHMT2, EI24, EIF2AK3, ELN, ERCC6, ERK2, ESCO2, ESR1, ESR2, EVC1, EVC2, EXT1, EXT2, F2, F5, FAM20C, FBLN1, FBLN5, FBN1, FBN2, FBXW8, FGF10, FGF21, FGF23, FGF8, FGF9, FGFR1, FGFR2, FGFR3, FGFR4, FGFRL1, FLNA, FLNB, FLT1, FOS, FUBP1, G6PC, GADD45G, GDF5, GH1, GH2, GHR, GHRH, GHRHR, GHSR, GJA1, GLG1, GLI2, GLI3, GLIS3, GNA12, GNAS, GNAS1, GNPAT, GNRH1, GNRH2, GNRHR, GPR161, GRB10, GRB2, GRHL2, GRK2, GSN, H19, HAPLN1, HDAC4, HESX1, HHIP, HMGA1, HMGA2, HNF1A, HNF1B, HOXA11, HOXD13, HRAS, HS3ST1, HSPG2, HUWE1, CHAD, CHRNA4, CHST3, CHSY1, IARS2, IDUA, IFT172, IFT80, IGF1, IGF1R, IGF2, IGF2BP1, IGFALS, IGFBP3, IGFBP4, IGSF1, IHH, IKBKG, IL11, INPPL1, INS, INSR, IRS1, IRS2, ITGA10, JAK2, JUN, KAL1, KDM5C, KLF4, KRAS, LBR, LEMD3, LFNG, LGALS3, LHX3, LHX4, LIFR, LMBR1, LMNA, LMX1B, LRP5, LTBP1, LTBP3, MAF, MATN1, MATN3, MESP2, METTL1, MGP, MIR140, MIR17, MIR18A, MIR19A, MIR19B1, MIR20A, MMP13, MMP14, MMP16, MMP2, MMP9, MSX2, MT1, MT2, MTHFR, NAB1, NAB2, NBAS, NCAPG, NFIX, NFKB1, NFKB2, NOG, NOS3, NPHS2, NPPC, NPR2, OBSL1, OSTM1, OTX2, P3H1, P63, PAPPA2, PAPSS2, PAX6, PAX8, PCNT, PEX7, PHEX, PI3K, PIAS1, PIAS3, PITX1, PITX2, PKDCC, PLAC1, PLAG1, PLAGL1, PLOD2, PLOD3, PNPLA6, POC1A, POLE, POR, POU1F1, PPARD, PPIB, PPP1R15B, PRG4, PRKAR1A, PRKG2, PRL, PRLR, PRMT7, PROKR2, PROP1, PRRX1, PRRX2, PTGS1, PTGS2, PTH, PTHLH, PTHR1, PTCH1	, PTPN1, PTPN11, PTPN2, PTPN3, PTPN6, PXMP3, RANKL, RBL1, RBL2, RBPJ, RDH11, RECQL, REV3L, RGS10, RIEG, RMRP, RNPC3, ROR2, RPS6, RUNX2, SALL1, SALL4, SBDS, SCMH1, SCUBE2, SCYL1BP1, SEDL, SERPINA3, SF1, SHC1, SHH, SHOX, SIK3, SIRP, SIX3, SKIV2L, SLC34A1, SLC34A3, SLC37A4, SLC39A13, SLC4A4, SMAD3, SMARCAL1, SOCS1, SOCS2, SOCS3, SOS1, SOST, SOX2, SOX3, SOX9, SRC, STAT1, STAT3, STAT4, STAT5, STAT5B, STK3, STK4, SUMO1,TBCE, TBX15, TBX19, TBX2, TBX3, TBX4, TBX5, TBXAS1, TCIRG1, TCPTP, TERT, TGFA, TGFB1, THRA, THRB, TITF1, TLE4, TM7SF2, TMEM16E, TNFRSF11A, TNFRSF11B, TNFSF11, TP53, TP63, TRHR, TRIP11, TRMT10A, TRPS1, TRPV4, TTC37, TWIST1, TWSG1, UBA2, UBP1, USP9X, VDR, WISP3, WNT3, WNT4, WNT7A, XBP1, XPA, XRCC4 ,ZBTB16, ZBTB38, ZMPSTE24, ZNF521
